# Controlled environment agriculture as urban infrastructure: advancing a public agenda for spatial equity

**DOI:** 10.1007/s44212-026-00109-y

**Published:** 2026-06-15

**Authors:** Xudong Rao, Azlan Zahid, Xinyue Ye, Devika Jain, Nick Duffield

**Affiliations:** 1 Department of Agricultural Economics, Texas A&M AgriLife Research, Texas A&M University System, Fort Worth, TX 76244 USA; 2 Department of Biological and Agricultural Engineering, Texas A&M AgriLife Research, Texas A&M University System, Dallas, TX 75252 USA; 3 Department of Geography and the Environment & ALA-GAINS (Alabama Geospatial AI & INformation Science) Hub & Alabama Center for the Advancement of AI, University of Alabama, Tuscaloosa, AL 35487 USA; 4 Center for Geographic Analysis, Harvard University, 1737 Cambridge Street, Cambridge, 02138 USA; 5 Department of Electrical and Computer Engineering & Texas A&M Institute of Data Science, Texas A&M University, College Station, TX 77840 USA

**Keywords:** Controlled environment agriculture, Urban food systems, Spatial justice, Public infrastructure, Sustainable planning

## Abstract

Controlled Environment Agriculture (CEA) has emerged as a high-tech solution to urban food security. However, its development has largely been driven by venture capital and profit-maximizing imperatives, resulting in limited CEA scalability, narrow crop diversity, and minimal engagement with issues of spatial equity—defined here as the fair distribution of food infrastructure across urban geographies. This Perspective calls for a paradigm shift: positioning CEA not as a standalone technological fix, but as a component of urban infrastructure that integrates into urban systems to deliver co-benefits for food justice, public health, and environmental resilience. We argue that the public value of CEA should be a central aim of CEA deployment, guiding both research and planning agendas. To advance this vision, we highlight the potential of Geospatial Artificial Intelligence (GeoAI) and High-Performance Computing (HPC) as underexplored tools to enable adaptive crop management, site-specific decision support, and identify underserved communities for targeted interventions. When combined with urban informatics approaches, these technologies can help embed CEA within inclusive, data-informed, and climate-resilient city systems. We conclude by outlining priorities for a public-interest CEA agenda that aligns with sustainable urban development and spatial justice.

## Introduction

Controlled Environment Agriculture (CEA), including greenhouses and indoor farms, represents a transformative evolution in food production. By employing technologies such as hydroponics, aeroponics, automation, and vertical farming, CEA enables crop cultivation in highly regulated environments. It has emerged as a promising spatial innovation at the intersection of agriculture, computer science, geographic information science, and urban planning. While CEA offers significant potential to improve efficiency, increase yields, and generate economic returns, much of its development and implementation to date has disproportionately prioritized corporate profitability over broader public benefits.

However, there is growing recognition that CEA’s potential can be reoriented within a more public-serving framework, defined here as a model that aligns agricultural innovation with societal goals such as food justice, climate resilience, and health equity. This framework positions CEA as urban infrastructure, meaning that it should be planned, funded, and governed as part of the essential systems that support city life, akin to transportation, housing, and water networks.

Urban stressors such as food insecurity, climate vulnerability, and health disparities disproportionately affect marginalized communities (Jansma & Wertheim-Heck, 2024). In this context, more equitable approaches to urban infrastructure should encompass both food justice, defined as the equitable distribution of nutritious, affordable, and culturally appropriate food, and spatial justice, which emphasizes the fair allocation of infrastructure and resources across geographic and demographic lines. Reframing CEA in this way highlights the often-overlooked non-monetary benefits that can far exceed its economic returns, thus aligning technological innovation with long-term community well-being and inclusive development.

CEA should be understood as part of a broader process of urban experimentation and institutional innovation. Urban experimentation involves testing new solutions through living labs, tactical urbanism, and cross-sector collaborations that emphasize participatory planning (Schreiber, 2025), while institutional innovation emphasizes collaborative approaches to infrastructure development (Almestar & Romero, 2025; Frantzeskaki et al., 2019). Positioning CEA within these paradigms situates it as a dynamic infrastructure shaped by social and spatial negotiations, rather than a static technological fix, and underscores its potential to advance resilience and equity agendas through iterative, collaborative processes.This perspective paper argues that the prevailing profit-centric approach fails to account for the multi-scalar, non-economic value of CEA, particularly in addressing structural vulnerabilities within urban food systems, health disparities, and ecological fragility. As cities increasingly face compound stressors, CEA should be repositioned not merely as a high-tech agribusiness, but as a planning-relevant tool to support community-scale nutrition, public health, and environmental justice agendas.

Cities are beginning to adopt this approach. In Ontario, Canada, a recent case study highlights how public–private partnerships and regional innovation ecosystems are enabling scalable CEA deployment to support food security and economic development. Similarly, Singapore’s “30 by 30” initiative aims to produce 30% of its nutritional needs locally by 2030, leveraging vertical farming and agri-tech to reduce import dependency and enhance urban resilience. These examples show that integrating CEA into urban systems requires policy alignment and community engagement.

Emerging technologies such as GeoAI and HPC can play a critical role in this reorientation by enabling more precise spatial analysis, predictive modeling, and equitable resource allocation. These tools support a shift from profit-centered models toward frameworks that prioritize spatially integrated, inclusive, and public-serving outcomes. Accordingly, we call for a redefinition of CEA research and policy that leverages technological advances to advance public-interest goals across urban systems. The essential technological, institutional, and spatial components of such a public-interest CEA model and their interactions are summarized in Table 1 and elaborated in the following sections.

**Table 1:** Public-interest CEA model—components and interactions

Component	Example Elements	Primary Roles	Interactions with Other Components
Technological	- CEA systems (vertical farms, greenhouses) - Geospatial AI (GeoAI) - High-Performance Computing (HPC) - Sensors & IoT	- Adaptive crop management - Site-specific decision support - Resource optimization	- Supplies data and analytics to institutions - Enables spatial targeting and dynamic management
Institutional	- Urban planning agencies - Public sector - Policy & regulatory frameworks - Community organizations	- Policy and regulation - Coordinated infrastructure planning - Public engagement	- Sets equity objectives and public value agendas - Directs and oversees technology deployment using spatial insights
Spatial	- Urban geography and land use - Food desert mapping - Infrastructure distribution - Equity/disparity analysis	- Identifies underserved communities - Site selection/prioritization - Monitors spatial equity	- Informs institutional targets for equity - Guides technological interventions for maximum impact

## Limitations of profit-driven CEA research

CEA was initially positioned as a response to urban food insecurity and resource stress, promising localized, high-efficiency food production (Calvo-Baltanás et al., 2025). Over time, however, the trajectory of CEA’s development has been predominantly shaped by market logics, particularly venture capital investment. Driven by rapid advances in automation, vertical farming architectures, AI-based monitoring systems, and modular hydroponic designs, CEA startups have been able to scale production and attract substantial private capital. These investment flows, in turn, have catalyzed technological innovation, leading to breakthroughs such as low-water cultivation methods and LED optimization for controlled environments. Despite these advancements, the prevailing profit-driven model for CEA continues to reveal several structural limitations across the entire value chain.

In its upstream segment, current CEA research and business operations are largely oriented toward short-cycle, high-yield crops, favoring rapid financial returns for investors at the expense of nutritional diversity for consumers (Janssen et al., 2019). Crops with greater nutritional or medicinal value, such as legumes and protein-dense crops, remain underfunded and under-researched due to their more complex environmental requirements and longer growth cycles (Calvo-Baltanás et al., 2025; Semba et al., 2021). Consequently, large-scale CEA operations primarily focus on producing leafy greens like lettuce and spinach, which are relatively easy to grow rapidly in confined spaces but offer limited micronutrient diversity (Harris et al., 2022). This persistent narrow crop focus, coupled with the dominance of large firms in research and development, undermines the long-term viability of the sector. Many of these businesses rely on widespread adoption of proprietary systems to validate market potential for investors. Yet, without inclusive feedback from a broader range of producers, technological innovation risks becoming stagnant and poorly adapted to varied urban and regional contexts. Ultimately, this model creates cyclical volatility and risks eventual obsolescence, underscoring the urgent need for a more distributed innovation ecosystem rooted in crop diversification and broader engagement with diverse technology users.

The midstream and downstream segments of the CEA industry also face several persistent obstacles. Because most CEA firms focus on premium leafy greens, these products are primarily marketed toward health-conscious, higher-income consumers shopping at upscale grocery chains or specialty markets while excluding both mid-income households and food-insecure populations. Major retailers such as Walmart, Kroger, and large-scale food service providers have the potential to expand consumer access to CEA products. However, these retailers typically require a diverse and consistent supply of produce as a baseline for entry, standards that most CEA operations currently struggle to meet. This ongoing disconnect between production capacity and market expectations continues to hinder broader integration of CEA into mainstream food systems. While some firms have attempted to locate facilities near existing distribution networks, these strategies are not always practical or financially viable, especially for smaller producers. Therefore, these constraints have prevented wide-scale adoption and limited consumer access, making it difficult for CEA growers to compete with conventional agriculture or sustain long-term business viability.

Altogether, the failure to diversify product offerings and customer bases not only makes individual CEA ventures more vulnerable to financial distress but also weakens investor confidence across the sector, contributing to the wave of investment withdrawals and downsized operations observed in recent years. Indeed, the CEA industry has seen several high-profile bankruptcies and many financially- strained operations during this period. *AeroFarms*, once considered a pioneer in vertical farming, filed for Chapter 11 bankruptcy in 2023, after struggling with high operational costs and declining investor confidence. *AppHarvest*, another major player in CEA and greenhouse farming, struggled with high debt, operational inefficiencies, and market saturation, which ultimately led to its bankruptcy filing. Eden Green Technology—a Texas-based controlled environment agriculture company with an annual production capacity reportedly in the millions of pounds of leafy greens—announced the closure of its operations in the Dallas-Fort Worth area, scheduled for December 2025. While specific details on Eden Green’s shutdown are not yet widely reported, sector-wide challenges such as high operational costs, scaling hurdles, and volatile access to capital are believed to have contributed to this sudden contraction. These setbacks are further exacerbated by a 91% drop in venture capital investments (Bradbury, 2023). Some public agencies and research institutions have redirected their funding and research priorities away from CEA technologies and back toward traditional sustainable agriculture. With the sharp decline in venture funding, fading investor enthusiasm, and shifting in priorities for public research funding, CEA risks losing momentum unless it adopts a broader, more inclusive mission.

To address these limitations, emerging digital tools such as GeoAI and HPC offer transformative potential. GeoAI enables fine-grained spatial analysis of urban food deserts, land use patterns, and demographic data, helping planners identify optimal sites for CEA deployment in underserved communities. HPC supports large-scale simulation of crop-environment interactions, enabling adaptive climate control and crop diversification strategies tailored to local conditions. When used in combination, these technologies can reorient CEA toward more inclusive access and enhanced ecological resilience.

From a critical urban studies standpoint, the current CEA paradigm exemplifies the structural tensions of contemporary urban infrastructure development. Rather than viewing CEA solely through technological or market-driven lenses, it should be understood as part of the wider political economy of urban space. Scholars of urban political ecology and spatial justice (Angelo & Wachsmuth, 2014; Gandy, 2022; Harvey, 2017; Heynen, 2013; Swyngedouw, 2004) emphasize that infrastructures are not neutral systems but socio-environmental assemblages shaped by power, capital, and uneven access to resources. Reframing CEA as a form of public urban infrastructure, embedded within debates on environmental justice and collective provisioning, shifts the focus to questions of who benefits from technological innovation and who remains excluded. In this light, the challenges of profit-driven CEA extend beyond technical efficiency or financial feasibility; they are fundamentally spatial and political. Thus, advancing CEA as a just form of urban infrastructure requires rethinking governance models, design priorities, and community participation to ensure that food production systems contribute to more equitable and ecologically grounded urban futures.

## Benefits of public-focused CEA research

High operational costs, market saturation, and declining investor confidence have exposed the limitations of a profit-first model in CEA. While CEA was initially celebrated as a disruptive innovation in agri-tech, many venture-backed CEA enterprises in the sector have struggled to reach profitability or scale in ways that serve broader public needs. The emphasis on yield efficiency, automation, and high-turnover crops has reinforced a narrow technological paradigm that overlooks the multidimensional value of CEA. In contrast, public-oriented CEA research and implementation offer the potential for broader and more durable impacts by enhancing nutrition and health status, improving environmental sustainability, and promoting equitable and resilient community development. By prioritizing long-term collective benefit over short-term investor returns, these approaches can reframe CEA not merely as a commercial technology, but as a public infrastructure essential to sustainable urban and regional futures.

### Nutrition and health

One of the most compelling arguments for prioritizing public-focused CEA research is its capacity to enhance nutrition and health outcomes for individuals, particularly those living in food deserts and climate-vulnerable regions. Scientific studies already indicate that leafy greens produced in controlled environments can exceed field-grown equivalents in micronutrients such as Vitamin C, beta-carotene, and antioxidants (Nekesa et al., 2025). Redirecting CEA research toward nutrient optimization and biofortification not only improves health outcomes but also reduces dependence on ultra-processed foods, thereby addressing the root causes of diet-related illnesses and chronic disease. Urban-based CEA allows produce to be harvested and consumed within hours, preserving nutritional value that is often lost in long-distance transportation. Such nutritional gains are consequential for communities lacking consistent access to fresh fruits and vegetables.

From a public health standpoint, CEA’s closed-loop nature also offers significant advantages. The ability to precisely control inputs such as light, humidity, nutrient delivery, and air quality reduces or eliminates the need for pesticides, herbicides, and fungicides. In addition, crops grown in CEA systems are also shielded from contaminated water sources and environmental pathogens, substantially lowering the risk of foodborne illnesses such as E. coli or Salmonella (Hamilton et al., 2023). These improvements contribute not only to heightened food safety but also to lower healthcare costs and reduced burden on public health systems.

### Environmental sustainability

Conventional agriculture, increasingly constrained by erratic weather patterns, soil degradation, and resource depletion, is becoming ill-equipped to ensure stable food supplies amid climate change. CEA, in contrast, offers year-round cultivation environments that are insulated from external shocks, thereby enabling more reliable and adaptive food production systems. By enabling local production close to consumers, CEA shortens supply chains, enhances freshness, and reduces logistical vulnerabilities, benefits that are especially crucial for marginalized communities with limited food access.

Despite these potential advantages, CEA’s environmental benefits are underleveraged under the current commercial paradigm, which often prioritizes output and efficiency over sustainability. Public-sector research has the potential to advance CEA as a low-impact, regenerative practice. This includes the development of ultra-efficient lighting systems, intelligent climate controls, closed-loop water systems, and renewable energy integration. When scaled through community-based initiatives, these innovations can support distributed food systems that are less dependent on long-distance transportation, thereby reducing emissions, improving local air quality, and enhancing regional food sovereignty (Gunapala et al., 2025; Janssen et al., 2019). By supporting localized, modular deployments, public-oriented CEA initiatives can also address challenges such as rural depopulation and urban blight, generating co-benefits across diverse locations.

### Community development

Beyond health and environmental outcomes, prioritizing public-focused CEA research also supports inclusive economic development. First, public-interest research can play a pivotal role in transforming CEA into a tool for nutritional equity. Investments in closed-loop irrigation systems, low-energy vertical farming, and renewable-integrated operations can yield context-sensitive, resource-efficient solutions suited to both dense urban cores and underserved rural areas. Moreover, expanding the crop portfolio in CEA systems opens possibilities for cultivating nutrient-dense and biofortified crops tailored to community-specific dietary needs. Community-based CEA programs can generate employment in farming, distribution, education, and ag-tech innovation, while enabling entrepreneurship among small-scale producers. These systems can anchor food cooperatives, social enterprises, and local business ecosystems. Additionally, integrating CEA programs into public schools, technical colleges, and research universities can cultivate a new generation of food system professionals trained in both sustainability science and digital agriculture. Extension programs and business incubators can also empower displaced rural workers, veterans, and underemployed youth to participate in the emerging green economy.

However, realizing these benefits requires more than biological or engineering advances; it demands the creation of new digital and analytical infrastructure. GeoAI facilitates the integration of multimodal data, including census demographics, health statistics, land use data, and environmental quality indicators, to identify spatial inequities in food access, optimize site selection for CEA facilities, and prioritize investment in underserved communities. GeoAI models can also incorporate satellite imagery, mobility patterns, and climate projections to simulate nutritional gaps and urban dietary landscapes in real time, offering planners actionable intelligence for addressing spatial injustice through food infrastructure. Simultaneously, HPC enables large-scale simulation of environmental dynamics within CEA systems, supporting adaptive climate control, crop modeling, and system optimization. By modeling how different crop varieties respond to microclimatic adjustments across varied contexts, HPC can guide the design of CEA systems suited to diverse geographies, from arid regions to dense metropolitan areas. Ultimately, the integrated application of GeoAI and HPC can reorient CEA away from a universal, investor-driven model toward context-sensitive, inclusive systems optimized for equity, resilience, and positive health outcomes.

Additionally, effective policy interventions must extend beyond simply increasing funding. Governments should support open-access data platforms, establish interagency partnerships between public health, environmental, and planning departments, and include CEA in comprehensive food system strategies (Guibrunet et al., 2025). Public procurement policies can create stable demand for CEA-grown produce in schools, hospitals, and emergency food programs. Moreover, grant mechanisms should prioritize projects that demonstrate measurable progress on health equity, sustainability, and community engagement, not solely technological novelty or yield metrics. In a pioneering initiative, the Dallas City Council adopted a comprehensive urban agricultural plan in 2023 as its long-term framework to guide current and future infrastructure planning efforts. Grounded in principles of mitigation, adaptation, environmental quality, and social equity principles, this plan includes ninety-seven actions across eight different focus areas (City of Dallas, 2023).

Municipal authorities and regional institutions can operationalize CEA as urban infrastructure by embedding it into local funding and planning frameworks. This strategy can involve dedicating resources through green infrastructure bonds, public grants (e.g., Singapore’s “30 by 30” express grants), or incentives (e.g., London’s Sustainable Drainage Action Plan) that support CEA adoption and long-term operations (Greater London Authority, 2016; Ministry of Sustainability & the Environment, 2020). Integrating CEA into zoning and land use policies—such as permitting it in residential, commercial, and industrial areas, or designating urban agriculture zones—ensures it becomes a recognized component of urban development. Additionally, cities and institutions can leverage procurement policies by prioritizing CEA-grown produce in schools (e.g., Gotham Greens for public schools in New York City) and hospitals (e.g., Rooftop farms of Boston Medical Center), thereby creating stable demand and aligning CEA deployment with broader public health and resilience goals (Gotham Greens, (n.d.); Boston Medical Center, (n.d.)). Through these mechanisms, authorities can mainstream CEA as part of essential city systems rather than treating it as a niche experiment.

In sum, CEA’s greatest potential lies not in maximizing shareholder returns, but in serving as a spatially embedded infrastructure that addresses the interconnected challenges of climate adaptation, health inequity, and food system resilience. Emerging tools such as GeoAI and HPC are not merely technical upgrades; they are critical enablers of spatial justice, enabling planners and policymakers to build more inclusive, adaptive, and responsive food systems. A reoriented CEA framework, centered on public good rather than private profit, offers a transformative opportunity to embed food equity, health promotion, and climate resilience into the urban fabric.

## Conclusion: advancing controlled environment agriculture through urban informatics

CEA can transform urban food systems by offering sustainable, resource-efficient responses to the interconnected challenges of food insecurity, climate change, and rapid urbanization. Beyond yield efficiency, CEA can also function as a critical public health intervention, delivering pesticide-free, nutrient-dense, and locally sourced food in urban settings increasingly affected by health disparities and environmental stressors. Yet despite its transformative potential, CEA research and implementation remain underfunded and narrowly focused on private-sector returns. To realize the full potential of CEA, a strategic shift is needed from private-sector models focused on profit to public-serving infrastructure that supports equity, health, and sustainability. This transition requires embedding CEA within urban informatics frameworks that guide planning, investment, and governance.

First, CEA should be redefined as public infrastructure that serves collective well-being rather than private profit. Public investment must emphasize inclusive access, crop diversity, and community-scale deployment, particularly in neighborhoods most affected by climate risks and food insecurity. Positioning CEA within existing climate adaptation plans and public health initiatives can help cities use food production as a tool for resilience, thereby reducing both environmental vulnerability and nutritional inequity.

Second, digital technologies such as GeoAI and HPC provide the analytical foundation for integrating CEA into broader urban systems. GeoAI can spatially identify food deserts, model population access, and optimize site selection to align CEA facilities with community needs and climate priorities. HPC, in turn, enables adaptive modeling of microclimates and energy demands, supporting efficient design and operation under changing environmental conditions.

Third, achieving systemic impact requires cross-sector policy integration. Governments should embed CEA into urban resilience and land-use plans, link it to public procurement programs (such as those for schools, hospitals, and shelters), and provide targeted incentives for deployment in underserved areas. Coordinated governance among planning, health, environmental, and agricultural agencies can ensure CEA supports multiple policy goals, advancing equity, food security, and climate resilience simultaneously.

Finally, future research and policy agenda must establish clear metrics for evaluating CEA’s contribution to health outcomes, spatial access, and adaptive capacity. Such indicators will be essential for demonstrating CEA’s value as a scalable, actionable infrastructure for building just, resilient, and climate-ready cities.

While this perspective advances a strong case for a public-interest orientation of CEA, it is important to acknowledge the complex interplay between public and private actors in shaping innovations in urban food systems. Tensions may arise as private firms continue to prioritize profitability and rapid technological scaling, sometimes at odds with public sector goals such as equity, broad accessibility, and long-term sustainability. Realizing a truly public-serving CEA agenda will require creative models of public–private partnership, clear accountability mechanisms, and policies that actively align market incentives with societal outcomes. By confronting these complexities directly, cities and policymakers can better harness CEA’s transformative potential as equitable, resilient urban infrastructure.

## Data Availability

Data sharing is not applicable to this article as no datasets were generated or analyzed during the current study.
